# Housing and the Cost of Land

**Published:** 1923-02

**Authors:** 


### Housing and the Cost of Land.

Sir,—In your December issue you ask if the cost of land is one of the obstacles to housing. I think you will find that, if it be an obstacle at all, it is so small as to be of no practical moment. Some years ago the London County Council published a statement of the cost of acquiring land for its housing schemes which showed that a very few pence per week per house was attributable to the purchase price of the land. The real difficulty now is the combined cost of labour and materials. B. H. T.

